# A Tri-Directional Examination of Parental Personality, Parenting Behaviors, and Contextual Factors in Influencing Adolescent Behavioral Outcomes

**DOI:** 10.1007/s10964-022-01602-8

**Published:** 2022-04-15

**Authors:** Tayler E. Truhan, Constantine Sedikides, Micheala McIlvenna, Lena Andrae, Rhiannon N. Turner, Kostas A. Papageorgiou

**Affiliations:** 1 School of Psychology, Queen’s University of Belfast, Belfast, UK; 2 Center for Research on Self and Identity, School of Psychology, University of Southampton, Southampton, UK

**Keywords:** Personality, Parenting, Adolescent behavior, Socioeconomic status, Adversity

## Abstract

Links between parental personality, parenting, and adolescent behavior have been well established. However, extant research is limited by the sole focus on parental Big Five personality, and not taking home and family context into account. These gaps were addressed in two studies. In study 1, context, parental personality, and their interactions were examined as predictors of parenting in separate mother and father models (parents only). In study 2, context, parental personality, and parenting were examined as predictors of adolescent behavioral outcomes (parent–adolescent dyads). Parents (*N* = 283, 45.6% mothers, *M*_*age*_ = 45.51 years) completed assessments of socioeconomic status (SES), adverse childhood experiences (ACEs), personality (Big Five, Dark Triad), and parenting. Adolescents (*N* = 257, 51.4% female, *M*_*age*_ = 13.65 years) completed an assessment of behavior. Parent Dark Triad domains explained more variance in parental warmth and hostility than the Big Five, but equivalent variance in adolescent behavior. SES interacted with maternal personality, whereas ACEs interacted with paternal personality, to predict parenting behavior. The results showcase the importance of assessing a wider spectrum of parental personality, and examining contextual factors, in affecting adolescent development.

## Introduction

Adolescent behavioral outcomes, including externalizing, internalizing, and prosocial behavior, are important markers of social well-being (Carlo & Padilla-Walker, 2020) as well as future mental health and psychopathology (Akingbuwa et al., 2020). The recently proposed Tri-Directional Framework of Parental Personality and Offspring Outcomes (Truhan et al., 2021) purports to organize the extensive body of literature specifying the pathways through which parental personality may influence offspring outcomes, such as behavior. In accordance with Process-Person-Context-Time (PPCT; Bronfenbrenner, 1995) and social learning (Bandura, 1977) models, the Tri-Directional Framework suggests that parental personality, parenting behavior, and contextual factors (e.g., socioeconomic status [SES]) play key roles in adolescent behavioral outcomes. Further, the Framework highlights that assessments of parental personality should extend beyond the traditional Big Five to encompass a wider spectrum of normative personality. However, there has been a lack of research that considers parental personality beyond the Big Five, and only one study to date has assessed the influence of context. The studies presented below address this gap in the literature by assessing parental Dark Triad personality domains, in addition to parenting behaviors and contextual factors, including SES and parents’ past adverse childhood experiences (ACEs), as influences on adolescent behavioral outcomes.

### Parental Personality, Parenting, and Context

Research on parental personality within the parent–offspring relationship typically focuses on the Big Five personality domains (Prinzie et al., 2009), comprising extraversion, agreeableness, neuroticism, conscientiousness, and openness (McCrae & Costa, 1996). Meta-analyses of parental personality and parenting behavior found that low neuroticism, but high extraversion, agreeableness, and conscientiousness had small but significant positive associations with parental warmth, adaptive control, and autonomy support (McCabe, 2014; Prinzie et al., 2009). In line with the Tri-Directional Framework, some scholars have suggested that the Big Five does not capture the full spectrum of normal personality (Feher & Vernon, 2021). The Dark Triad of personality is a relatively understudied model as applied to the parent–offspring relationship, yet it may be useful in explaining parenting behaviors and offspring outcomes.

The Dark Triad is a cluster of three interrelated, yet distinct, personality domains: subclinical narcissism, Machiavellianism (e.g., manipulation, detachment), and subclinical psychopathy (e.g., lack of empathy, antisociality; Paulhus & Williams, 2002). In parenting research, parental narcissism is positively associated with both greater parental warmth via grandiose narcissism (Dottan & Cohen, 2014), measured as the total Narcissistic Personality Inventory (NPI; Raskin & Terry, 1988) score, and greater authoritarian (i.e., highly controlling) parenting via maladaptive narcissism (the mean score of the entitlement, exploitativeness, and exhibitionism NPI facets; Hart et al., 2017). Parental control may consist of both adaptive and maladaptive aspects of parenting, depending on the degree of regulation, manipulation, and management implicated in the parent–offspring relationship. In the current studies, control was measured along a continuum of low/lax control (i.e., youth are allowed to fully regulate their own activities) to strict/restrictive control (i.e., parents almost always try to control their child’s behavior). Generally, a moderate amount of behavioral control, in which the parent is flexible in their control in some contexts but firm in others, is considered adaptive for youth (Rohner et al., 2005).

Therefore, narcissism may be associated with parenting that is adaptive (i.e., warmth) or maladaptive (i.e., high control) for youth depending on the specific parental narcissistic traits assessed. Whereas parental Machiavellianism and psychopathy have been solely linked with negative parenting behaviors (Krupić et al., 2020; Láng, 2018). Structural analyses of narcissism indicate that narcissism consists of three major traits, identified as agentic extraversion (e.g., assertiveness), antagonism (e.g., exploitation), and narcissistic neuroticism (e.g., need for admiration; Truhan et al., 2021). The current studies will extend previous literature by examining associations between specific narcissistic traits, parenting behaviors, and adolescent outcomes to identify which traits function adaptively or maladaptively.

Examination of personality in context has shown that typical associations between personality and parenting behavior vary according to environmental stress (Kochanska et al., 2012). Personality traits influence individuals’ responses to adversity and stressful environments (Connor-Smith & Flachsbart, 2007). Also, contextual factors, including SES and parents’ ACEs, impact offspring development (Bradley & Corwyn, 2002; Felitti et al., 1998). As in the PPCT model, SES and parents’ ACEs are important components to an individual’s immediate home (i.e., SES) and family (i.e., parents’ ACEs) context, and will impact continual processes of development (i.e., parenting behavior) over time. One study that examined family demographic risk—defined as parental education level, age, and family income—reported that, for mothers who were low on extraversion, high demographic risk predicted greater maternal power assertion (Kochanska et al., 2007). ACEs have been linked to a range of future negative life outcomes, including physical and mental health problems (Chapman et al., 2004). Relevant findings indicate that SES and parents’ ACEs are influential to adolescent outcomes as a potential source of stress for parents, and may moderate certain associations between parental personality and parenting behavior.

### Parental Personality, Parenting, and Adolescent Behavior

In addition to assessing parenting behavior as an outcome in Study 1, parental personality and parenting behavior were assessed as predictors of adolescent externalizing, internalizing, and prosocial behavior in Study 2. According to social learning theory (Bandura, 1977), offspring model their behavior on that of their parents. As such, adolescents who experience warm and supportive parenting may model this prosocial behavior in their interactions with other individuals. However, hostile and aggressive parenting may engender externalizing behaviors in offspring. In a longitudinal study with mothers, fathers, and adolescents, both maternal and paternal warmth were positively associated with adolescent prosocial behavior, whereas only paternal hostility was negatively associated with adolescent prosocial behavior (Padilla-Walker et al., 2016).

In terms of parental Big Five personality, parental conscientiousness, especially in mothers, directly and indirectly, through ease of setting limits, predicts lower adolescent externalizing behavior problems (Oliver et al., 2009). Low parental agreeableness is especially salient in predicting more severe offspring externalizing behaviors (Krupić et al., 2020). In a study with young children aged two to six, maternal conscientiousness and agreeableness were negatively associated with child internalizing behavior problems, whereas punitive parenting (e.g., aggression, hostility, yelling) was positively associated with child internalizing (Puff & Renk, 2016).

In terms of parental Dark Triad personality, one study reported that negative parenting behaviors fully mediated the relationship between parental subclinical narcissism and adolescent symptoms of depression and anxiety in both mother and father models, except that putdown/shaming was not significant for fathers, and low care (e.g., lack of empathy) was not significant for mothers (Dentale et al., 2015). In a study with fathers and their offspring, higher paternal narcissism was associated with increased involvement with their adolescent children (Dottan & Cohen, 2014). Also, maternal psychopathy was positively associated with externalizing problems in pre-adolescent boys (Robinson et al., 2016).

No studies to date have assessed parental Machiavellianism or specific narcissistic traits (e.g., antagonism) in relation to adolescent behavior. As these domains capture aspects of interpersonal behavior that may be maladaptive, or potentially adaptive in the case of narcissism, they were examined in association with parenting behavior and adolescent development in the current studies. Prior work also highlights the differential influence that maternal and paternal personality traits exert on parenting styles and offspring outcomes, such as the difference in paternal and maternal narcissism (Dentale et al., 2015). Therefore, in Study 1 models were run separately based on parent gender.

## The Current Studies

Although consistent links have been demonstrated between parental personality, parenting, and offspring outcomes, there have been a lack of studies which examine personality beyond the Big Five. Further, only one study to date has examined the influence of contextual factors on these associations. The current studies examine comprehensively the associations among parental personality, parenting behavior, context, and adolescent behavioral outcomes. In Study 1, SES (i.e., a composite score of household income, educational attainment, age at birth of the child, and single parent status) and parents’ ACEs were included as moderators of the personality-parenting relationship in separate mother and father models. It is expected that, of the Big Five domains, extraversion, agreeableness, and conscientiousness will show positive associations with adaptive parenting, whereas neuroticism will be negative; of the Dark Triad domains, that agentic extraversion will be positively associated with adaptive parenting, whereas antagonism, narcissistic neuroticism, psychopathy and Machiavellianism will be negative (Hypothesis 1). Based on preliminary research which found that ecological adversity moderated maternal Big Five personality-parenting associations, it is hypothesized that SES and parents’ ACEs will moderate personality-parenting associations for both Big Five and Dark Triad models (Hypothesis 2). Last, it is expected that associations between parental personality and adolescent behavioral outcomes will parallel associations between personality and parenting in terms of being adaptive or maladaptive (e.g., agentic extraversion will be associated with adaptive parenting and adolescent behavioral strengths; Hypothesis 3).

## Methods

### Sample and Procedure

Parents and adolescents were recruited via secondary schools throughout Northern Ireland and the online platform Prolific Academic. Parents and adolescents responded to online self-report questionnaires, after providing consent or assent (age 11–15) to take part in the study. Participants were 283 parents with a mean age of 45.51 years (*Range* = 28–63, *SD* = 6.93) and 257 adolescents with a mean age of 13.65 years (*Range* = 11–17, *SD* = 1.96). Of parents, 45.6% were mothers, whereas, of adolescents, 51.4% were female. Parents were 84.1% White Irish/British, 10.2% White European, 2.5% Asian, 1.4% mixed ethnicities; one participant identified as Black African, another as Black Caribbean, and a third as “Other”. Three parents had more than one child, and therefore completed questionnaires for each of their participating children. This study was conducted as part of the Parents and Children Together (PaCT) Project, which was approved by the Faculty Research Ethics Committee of Queen’s University Belfast (Reference No: EPS 18_190). Power analysis conducted with G*Power 3.1 (Faul et al., 2009) indicated that this study had the power to detect small to large interaction effects (f^2^ ≥ 0.11; required sample size 121) for mothers, but not very small to small interaction effects (f^2^ ≤ 0.10; required sample size 132 or greater). For fathers, this study did not have the power to detect very small to small interaction effects (f^2^ ≤ 0.08; required sample size 165 or greater).

### Measures

#### Parental personality

A range of parental personality domains were assessed with the Big Five Inventory (BFI; John et al., 1991), Dirty Dozen (DD; Jonason & Webster, 2010), and Five Factor Narcissism Inventory Short Form (FFNI-SF; Sherman et al., 2015). Narcissism was assessed with the FFNI-SF, and Machiavellianism/psychopathy with the DD. The BFI is a 44-item inventory that assesses the Big Five factors of personality, including extraversion versus introversion, agreeableness versus antagonism, conscientiousness versus lack of direction, openness versus closedness to experience, and neuroticism versus emotional stability (*All Range* = 1–5). A sample agreeableness item is: “Is helpful and unselfish with others”. The DD is a 12-item self-report measure of Machiavellianism, subclinical narcissism, and subclinical psychopathy (*All Range* = 4–20). A sample Machiavellianism item is: “I have used deceit or lied to get my way”. The FFNI-SF is a 60-item questionnaire that assesses three higher order factors of narcissism: antagonism (*Range* = 32–160), agentic extraversion (*Range* = 16–80), and narcissistic neuroticism (*Range* = 12–60). A sample antagonism item is: “I have at times gone into a rage when not treated rightly”. Scores are averaged across items for the BFI, and summed across items for the FFNI and DD. All personality assessments were rated on a Likert scale from 1–5 (1 = *disagree strongly*, 5 = *agree strongly*). Reliability statistics for all included measures are presented in Table 1.

**Table 1:** Descriptive statistics and reliability for all scales

Variable	Mean (*SD*)	Variance	Median	Range	Kurtosis	Skew	Items (*N*)	*ω*
*Parents* (*N* = 283)								
ACE	1.45 (*2.12*)	4.49	1.00	10.00	3.60	1.92	10	0.84
SES	17.89 (*4.42*)	19.50	18.00	21.00	−0.64	−0.03	4	0.57
AGR	3.87 (*0.53*)	0.29	3.89	3.22	0.46	−0.44	9	0.78
CSC	3.96 (*0.58*)	0.34	4.00	3.00	0.26	−0.41	9	0.85
EXT	3.19 (*0.74*)	0.55	3.25	3.75	−0.47	−0.22	8	0.87
NER	2.65 (*0.79*)	0.63	2.63	3.88	−0.49	0.27	8	0.88
OPN	3.37 (*0.56*)	0.31	3.30	3.70	0.61	−0.02	10	0.81
MAC	7.74 (*3.18*)	10.11	7.00	15.00	−0.31	0.64	4	0.78
PSY	7.28 (*3.17*)	10.05	7.00	16.00	1.38	1.21	4	0.85
ATG	65.24 (*16.04*)	257.31	63.00	84.00	−0.02	0.52	32	0.90
AGE	42.72 (*10.57*)	111.64	42.00	61.00	−0.07	0.25	16	0.87
NNR	35.49 (*8.08*)	65.36	36.00	43.00	−0.07	0.10	12	0.83
HTY	7.59 (*1.94*)	3.78	7.00	10.00	−1.44	0.23	6	0.68
WRM	30.15 (*2.37*)	5.61	31.00	11.00	−1.43	−0.22	8	0.78
CTL	14.24 (*2.17*)	4.70	14.00	13.00	0.56	−0.47	5	0.52
*Adolescents* (*N* = 257)								
EPB	5.92 (*3.83*)	14.65	5.00	17.00	−0.43	0.49	10	0.79
IPB	5.17 (*3.33*)	11.09	5.00	16.00	0.04	0.69	10	0.72
PRO	7.57 (*1.93*)	3.73	8.00	8.00	−0.33	−0.57	5	0.74

*Note. ω* = McDonald’s omega (McDonald, 1999). Parent Variables: *AGR* agreeableness, *CSC* conscientiousness, *EXT* extraversion, *NER* neuroticism, *OPN* Openness, *MAC* Machiavellianism, *PSY* psychopathy, *ATG* antagonism, *AGE* agentic extraversion, *NNR* narcissistic neuroticism, *HTY* hostility, *WRM* warmth, *CTL* control. Adolescent Variables: *EPB* externalizing problem behavior, *IPB* internalizing problem behavior, *PRO* prosocial behavior

#### Parenting

Parents self-reported on warmth (e.g., care, affection; *Range* = 8–32), hostility (e.g., irritability, anger; *Range* = 6–24), and behavioral control (e.g., permissiveness-strictness; *Range* = 5–20) with the Parental Acceptance–Rejection Questionnaire/Control—Short Form (Parent PARQ/Control; Rohner et al., 2005). The PARQ/Control Short Form consists of 29 items. A sample warmth item is: “I make it easy for my child to confide in me” (1 = *almost never true*, 4 = *almost always true*). Scores are summed across items. Scores at or below the midpoint of the warmth scale indicate more coldness than warmth. The opposite is true for the hostility and control scales.

#### Context

Contextual factors were assessed with the Adverse Childhood Experiences Questionnaire (ACE-Q; Felitti et al., 1998) as well as a demographics measure relevant to SES (adapted from Kochanska et al., 2012). The ACE-Q is a 10-item self-report measure that identifies experiences of abuse and neglect via a checklist of adverse life events, generating a total ACE score (*Range* = 0–10). To create a total SES score, parents answered questions on self and partner income (1 = Less than 6000 GBP, 2 = 6000 to less than 13,000 GBP, 3 = 13,000 to less than 19,000 GBP, 4 = 19,000 to less than 26,000 GBP, 5 = 26,000 to less than 32,000 GBP, 6 = 32,000 to less than 48,000 GBP, 7 = 48,000 to less than 64,000 GBP, 8 = 64,000 GBP or more), age at birth of the child (0 = 19 or younger, 1 = 20–22 years of age, 2 = 23–25 years of age, 3 = 26 or older), educational attainment (1 = No formal schooling, 2 = primary school, 3 = GCSEs, 4 = A Levels, 5 = Advanced Diploma, 6 = Undergraduate Degree, 7 = Postgraduate Degree), and single parent status (1 = single parent, 2 = two-parent household). Higher scores indicate higher SES (*Range* = 4–28).

#### Adolescent behavioral outcomes

Adolescent self-reported behavioral outcomes were assessed with the Strengths and Difficulties Questionnaire (SDQ; Goodman, 1997). The SDQ is a 25-item self-report measure assessing internalizing and externalizing behaviors (*Range* = 0–20), including emotional symptoms, peer relationship problems, conduct problems, and hyperactivity, as well as prosocial behavior (*Range* = 0–10). A sample externalizing behavior item is: “I take things that are not mine from home, school, or elsewhere” (0 = *not true*, 1 = *sometimes true*, 2 = *certainly true*).

### Statistical Analyses

Prior to conducting the main analyses, all variables were checked for missing or mis-coded data and normality. No data was mis-coded. There were 11 cases of missing data for continuous variables. Little’s (1988) test of missing values was used to determine if data was missing completely at random. Little’s Missing Completely at Random (MCAR) test showed data were missing completely at random: *χ*^2^(68) = 53.13, *p* = 0.91. Therefore, missing data points were imputed using the expectation maximization algorithm. Parental warmth and hostility were negatively and positively skewed, respectively. Therefore, these variables were transformed using Rankit’s formula (Bliss et al., 1956), as this rank-based normalization method outperforms other common methods (e.g., Van der Waerden method; Solomon & Sawilowsky, 2009).

#### Study 1

Hierarchical multiple regression models (Aiken & West, 1991) were carried out via the R Statistical Software package (R Core Team, 2020) to examine SES and ACEs, parental personality, and their interactions as predictors of parental warmth, hostility, and control. Predictors were entered as follows: SES and ACEs in Step 1, personality traits in Step 2, and interactions (Personality × SES, Personality × ACEs) in Step 3. Regression models were examined separately for fathers (*N* = 154) and mothers (*N* = 129), and are presented in Tables 2–5. Only significant interactions were included. Interaction effects identified in the hierarchical regression models of Study 1 were tested separately, and the Johnson–Neyman technique (Johnson & Neyman, 1936) and interaction plots were used to examine significant interaction effects. Rather than methods that use fixed values of the moderator, such as simple slopes (Aiken & West, 1991), the Johnson–Neyman procedure solves for the value of the moderator at which the relation between the predictor and criterion variables achieves significance.

**Table 2:** Paternal big five domains and contextual factors as predictors of parenting

	Warmth			Hostility			Control		
Predictors	*B*	SE *B*	*β*	*B*	SE *B*	*β*	*B*	SE *B*	*β*
SES	0.00	0.01	0.02	0.01	0.01	0.15	0.03	0.05	0.05
ACEs	−0.02	0.01	−0.14	0.02	0.01	0.15	0.13	0.10	0.11
*Step 1*	*R*^*2*^ = 0.02			*R*^*2*^ = 0.04			*R*^*2*^ = 0.01		
	*F*(2, 151) = 1.68, *ns*			*F*(2, 151) = 2.98, *ns*			*F*(2, 151) = 0.97, *ns*		
SES	0.00	0.01	0.00	0.01	0.01	0.16*	0.03	0.05	0.05
ACEs	−0.01	0.01	−0.09	0.01	0.01	0.08	0.09	0.10	0.07
Ext	0.02	0.03	0.05	0.01	0.03	0.04	0.13	0.23	0.05
Agr	0.08	0.04	0.18*	−0.14	0.04	−0.31***	−0.48	0.33	−0.13
Csc	0.07	0.04	0.17	0.00	0.04	0.00	0.72	0.30	0.22*
Ner	−0.02	0.04	−0.06	0.04	0.03	0.12	0.44	0.27	0.16
Opn	0.01	0.04	0.01	−0.04	0.04	−0.10	−0.71	0.30	−0.20*
*Step 2*	*R*^*2*^ = 0.13			*R*^*2*^ = 0.19			*R*^*2*^ = 0.11		
	*F*(7, 146) = 3.16**			*F*(7, 146) = 5.10***			*F*(7, 146) = 2.48*		
	*F*∆(5, 146) = 4.15**			*F*∆(5, 146) = 5.50***			*F*∆(5, 146) = 3.01*		
SES	0.00	0.01	0.02	0.01	0.01	0.15	0.04	0.05	0.06
ACEs	−0.01	0.01	−0.09	0.02	0.01	0.10	0.13	0.11	0.10
Ext	0.03	0.03	0.08	0.01	0.03	0.02	0.14	0.24	0.05
Agr	0.05	0.04	0.11	−0.14	0.04	−0.31***	−0.40	0.34	−0.11
Csc	0.1	0.04	0.22*	−0.01	0.04	−0.02	0.70	0.32	0.21*
Ner	−0.02	0.04	−0.05	0.04	0.04	0.11	0.44	0.28	0.16
Opn	0.00	0.04	0.00	−0.04	0.04	−0.08	−0.78	0.31	−0.22*
Csc x ACEs	−0.06	0.02	−0.28**						
*Step 3*	*R*^*2*^ = 0.24			*R*^*2*^ = 0.22			*R*^*2*^ = 0.16		
	*F*(17, 136) = 2.59**			*F*(17, 136) = 2.21**			*F*(17, 136) = 1.48, *ns*		
	*F*∆(10, 136) = 1.92*			*F*∆(10, 136) = 0.35, *ns*			*F*∆(10, 136) = 0.80, *ns*		

*Note*. Presented values are from all steps of the hierarchical regression analyses with fathers (*N* = 154). Predictors were entered as follows: Step 1: SES and ACEs, Step 2: Paternal Big Five domains, Step 3: The five interaction terms of SES and Big Five and five interaction terms of ACEs and Big Five. Only significant interactions are included. *Ext* extraversion, *Agr* agreeableness, *Csc* conscientiousness, *Ner* neuroticism, *Opn* openness

*p < 0.05, **p < 0.01, ***p < 0.001

**Table 3:** Maternal big five domains and contextual factors as predictors of parenting

	Warmth			Hostility			Control		
Predictors	*B*	SE *B*	*β*	*B*	SE *B*	*β*	*B*	SE *B*	*β*
SES	0.00	0.00	0.09	0.01	0.00	0.12	0.08	0.04	0.20*
ACEs	0.00	0.01	−0.04	0.02	0.01	0.17	0.12	0.07	0.14
*Step 1*	*R*^*2*^ = 0.01			*R*^*2*^ = 0.04			*R*^*2*^ = 0.05		
	*F*(2, 126) = 0.74, *ns*			*F*(2, 126) = 2.42, *ns*			*F*(2, 126) = 3.41*		
SES	0.00	0.00	0.06	0.01	0.00	0.16	0.07	0.04	0.17
ACEs	0.00	0.01	−0.03	0.01	0.01	0.13	0.11	0.08	0.13
Ext	−0.05	0.05	−0.13	0.03	0.05	0.06	−0.31	0.43	−0.10
Agr	0.12	0.06	0.20*	−0.22	0.06	−0.35***	−0.86	0.47	−0.18
Csc	0.05	0.06	0.09	−0.04	0.06	−0.07	0.63	0.48	0.14
Ner	−0.03	0.04	−0.10	0.04	0.04	0.12	−0.17	0.32	−0.06
Opn	0.04	0.06	0.08	−0.01	0.06	−0.01	0.28	0.46	0.06
*Step 2*	*R*^*2*^ = 0.09			*R*^*2*^ = 0.21			*R*^*2*^ = 0.08		
	*F*(7, 121) = 1.62, *ns*			*F*(7, 121) = 4.67***			*F*(7, 121) = 1.57, *ns*		
	*F*∆(5, 121) = 2.01			*F*∆(5, 121) = 5.26***			*F*∆(5, 121) = 0.86, *ns*		
SES	0.00	0.00	0.01	0.01	0.01	0.14	0.07	0.04	0.17
ACEs	0.00	0.01	−0.01	0.01	0.01	0.10	0.11	0.09	0.13
Ext	−0.04	0.05	−0.09	0.04	0.06	0.09	−0.14	0.44	−0.04
Agr	0.13	0.06	0.22*	−0.22	0.06	−0.34***	−0.93	0.47	−0.20
Csc	0.04	0.06	0.07	−0.03	0.06	−0.05	0.52	0.49	0.12
Ner	−0.01	0.04	−0.05	0.04	0.04	0.12	−0.03	0.35	−0.01
Opn	0.04	0.06	0.08	−0.05	0.06	−0.08	0.36	0.49	0.08
Ext x SES							0.18	0.08	0.28*
*Step 3*	*R*^*2*^ = 0.18			*R*^*2*^ = 0.26			*R*^*2*^ = 0.17		
	*F*(17, 111) = 1.46, *ns*			*F*(17, 111) = 2.29**			*F*(17, 111) = 1.35, *ns*		
	*F*∆(10, 111) = 0.79, *ns*			*F*∆(10, 111) = .70, *ns*			*F*∆(10, 111) = 1.17, *ns*		

*Note*. Presented values are from all steps of the hierarchical regression analyses with mothers (*N* = 129). Predictors were entered as follows: Step 1: SES and ACEs, Step 2: Maternal Big Five domains, Step 3: The five interaction terms of SES and Big Five and five interaction terms of ACEs and Big Five. Only significant interactions are included. *Ext* extraversion, *Agr* agreeableness, *Csc* conscientiousness, *Ner* neuroticism, *Opn* openness

*p < 0.05, **p < 0.01, ***p < 0.001

**Table 4:** Paternal dark triad domains and contextual factors as predictors of parenting

	Warmth			Hostility			Control		
Predictors	*B*	SE *B*	*β*	*B*	SE *B*	*β*	*B*	SE *B*	*β*
SES	0.00	0.01	0.02	0.01	0.01	0.15	0.03	0.05	0.05
ACEs	−0.02	0.01	−0.14	0.02	0.01	0.15	0.13	0.10	0.11
*Step 1*	*R*^*2*^ = 0.02			*R*^*2*^ = 0.04			*R*^*2*^ = 0.01		
	*F*(2, 151) = 1.68, *ns*			*F*(2, 151) = 2.98, *ns*			*F*(2, 151) = 0.97, *ns*		
SES	0.00	0.01	0.03	0.01	0.01	0.15	0.02	0.05	0.04
ACEs	−0.01	0.01	−0.03	0.01	0.01	0.06	0.11	0.11	0.08
Psy	−0.01	0.01	−0.17	0.02	0.01	0.28*	0.10	0.08	0.16
Mac	−0.01	0.01	−0.13	0.00	0.01	0.00	0.01	0.07	0.01
NNe	−0.01	0.00	−0.21**	0.00	0.00	0.15	0.03	0.02	0.11
Ant	0.00	0.00	−0.12	0.00	0.00	0.14	−0.02	0.02	−0.13
AgE	0.00	0.00	0.14	0.00	0.00	−0.15	0.00	0.02	−0.01
*Step 2*	*R*^*2*^ = 0.19			*R*^*2*^ = 0.21			*R*^*2*^ = 0.04		
	*F*(7, 146) = 4.93***			*F*(7, 146) = 5.43***			*F*(7, 146) = 0.83, *ns*		
	*F*∆(5, 146) = 6.53***			*F*∆(5, 146) = 6.33***			*F*∆(5, 146) = 0.75, *ns*		
SES	0.00	0.01	0.01	0.01	0.01	0.13	0.01	0.05	0.01
ACEs	−0.03	0.02	−0.19*	0.02	0.01	0.11	0.05	0.13	0.04
Psy	−0.01	0.01	−0.12	0.02	0.01	0.26*	0.09	0.08	0.14
Mac	−0.01	0.01	−0.12	0.00	0.01	−0.02	0.04	0.08	0.06
NNe	−0.01	0.00	−0.19*	0.00	0.00	0.11	0.02	0.02	0.09
Ant	0.00	0.00	−0.22	0.00	0.00	0.21	−0.02	0.02	−0.17
AgE	0.01	0.00	0.21*	−0.01	0.00	−0.20*	0.00	0.02	−0.02
Mac x ACEs	0.02	0.05	0.39***						
*Step 3*	*R*^*2*^ = 0.29			*R*^*2*^ = 0.28			*R*^*2*^ = 0.08		
	*F*(17, 136) = 3.32***			*F*(17, 136) = 3.06***			*F*(17, 136) = 0.66, *ns*		
	*F*∆(10, 136) = 1.97*			*F*∆(10, 136) = 1.32, *ns*			*F*∆(10, 136) = 0.55, *ns*		

*Note*. Presented values are from all steps of the hierarchical regression analyses with fathers (*N* = 154). Predictors were entered as follows: Step 1: SES and ACEs, Step 2: Paternal Dark Triad domains, Step 3: The five interaction terms of SES and Dark Triad and five interaction terms of ACEs and Dark Triad. Only significant interactions are included. *Psy* psychopathy, *Mac* machiavellianism, *NNe* narcissistic neuroticism, *Ant* antagonism, *AgE* agentic extraversion

*p < 0.05, **p < 0.01, ***p < 0.001

**Table 5:** Maternal dark triad domains and contextual factors as predictors of parenting

	Warmth			Hostility			Control		
Predictors	*B*	SE *B*	*β*	*B*	SE *B*	*β*	*B*	SE *B*	*β*
SES	0.00	0.00	0.09	0.01	0.00	0.12	0.08	0.04	0.20*
ACEs	0.00	0.01	−0.04	0.02	0.01	0.17	0.12	0.07	0.14
*Step 1*	*R*^*2*^ = 0.01			*R*^*2*^ = 0.04			*R*^*2*^ = 0.05		
	*F*(2, 126) = 0.74, *ns*			*F*(2, 126) = 2.42, *ns*			*F*(2, 126) = 3.41*		
SES	0.00	0.00	0.04	0.01	0.00	0.13	0.09	0.04	0.21*
ACEs	0.00	0.01	−0.05	0.00	0.01	0.02	0.07	0.08	0.09
Psy	0.00	0.01	0.00	0.00	0.01	−0.01	0.02	0.11	0.02
Mac	−0.01	0.01	−0.10	0.04	0.01	0.34**	0.13	0.11	0.15
NNe	0.00	0.00	0.09	0.01	0.00	0.18*	0.02	0.03	0.07
Ant	0.00	0.00	−0.22	0.00	0.00	0.15	0.00	0.02	−0.02
AgE	0.00	0.00	0.15	0.00	0.00	−0.05	−0.02	0.02	−0.07
*Step 2*	*R*^*2*^ = 0.07			*R*^*2*^ = 0.25			*R*^*2*^ = 0.08		
	*F*(7, 121) = 1.27, *ns*			*F*(7, 121) = 5.85***			*F*(7, 121) = 1.51, *ns*		
	*F*∆(5, 121) = 1.61, *ns*			*F*∆(5, 121) = 7.00***			*F*∆(5, 121) = 0.74, *ns*		
SES	0.00	0.00	−0.01	0.01	0.01	0.12	0.07	0.04	0.17
ACEs	−0.01	0.01	−0.06	0.00	0.01	−0.01	0.03	0.10	0.04
Psy	0.00	0.01	0.02	−0.01	0.01	−0.04	−0.01	0.12	−0.01
Mac	−0.02	0.01	−0.18	0.05	0.01	0.40***	0.14	0.12	0.16
NNe	0.01	0.00	0.18	0.00	0.00	0.12	0.02	0.03	0.06
Ant	−0.01	0.00	−0.29*	0.00	0.00	0.15	−0.01	0.02	−0.05
AgE	0.01	0.00	0.19	0.00	0.00	−0.06	−0.02	0.03	−0.07
*Step 3*	*R*^*2*^ = 0.22			*R*^*2*^ = 0.32			*R*^*2*^ = 0.13		
	*F*(17, 111) = 1.79*			*F*(17, 111) = 3.01***			*F*(17, 111) = 0.95, *ns*		
	*F*∆(10, 111) = 2.07*			*F*∆(10, 111) = 1.02, *ns*			*F*∆(10, 111) = 0.59, *ns*		

*Note*. Presented values are from all steps of the hierarchical regression analyses with mothers (*N* = 129). Predictors were entered as follows: Step 1: SES and ACEs, Step 2: Maternal Dark Triad domains, Step 3: The five interaction terms of SES and Dark Triad and five interaction terms of ACEs and Dark Triad. No significant interactions were found. *Psy* psychopathy, *Mac* machiavellianism, *NNe* narcissistic neuroticism, *Ant* antagonism, *AgE* agentic extraversion

*p < 0.05, **p < 0.01, ***p < 0.001

#### Study 2

Hierarchical multiple regression models were carried out to examine adolescent age and sex, SES, parents’ ACEs, parental personality, and parenting behavior as predictors of adolescent self-reported behavioral outcomes. Predictors were entered as follows: adolescent age and sex, SES and parents’ ACEs in Step 1, parental personality in Step 2, and parenting behaviors in Step 3. Regression models were conducted with the total parent–adolescent dyad sample (*N* = 257 dyads), and are presented in Tables 6 and 7. Separate models were not examined for mothers and fathers in Study 2 as these models contained more direct predictors and would not have enough power if separated by parent gender. Analyses with G*Power 3.1 indicated required sample size to detect a small–medium effect (f^2^ = 0.11) with 13 predictors (total predictors in Step 3) was 253.

**Table 6:** Parent big five, context, and parenting as predictors of adolescent behavioral outcomes

	Externalizing			Internalizing			Prosocial		
Predictors	*B*	SE *B*	*β*	*B*	SE *B*	*β*	*B*	SE *B*	*β*
A. Sex	−0.31	0.48	−0.04	1.45	0.41	0.22***	0.58	0.23	0.15*
A. Age	0.15	0.12	0.08	0.08	0.10	0.05	−0.02	0.06	−0.02
P. Gender	0.15	0.49	0.02	−0.04	0.41	−0.01	0.77	0.24	0.20**
SES	−0.05	0.05	−0.06	−0.06	0.05	−0.08	0.05	0.03	0.12*
ACEs	0.22	0.11	0.12	0.14	0.10	0.09	−0.02	0.06	−0.02
*Step 1*	*R*^*2*^ = 0.02			*R*^*2*^ = 0.07			*R*^*2*^ = 0.08		
	*F*(5, 251) = 1.28, *ns*			*F*(5, 251) = 3.81**			*F*(5, 251) = 4.26***		
A. Sex	−0.34	0.48	−0.04	1.40	0.41	0.21***	0.68	0.23	0.18**
A. Age	0.21	0.12	0.11	0.12	0.11	0.07	−0.02	0.06	−0.03
P. Gender	−0.05	0.54	−0.01	−0.25	0.46	−0.04	0.43	0.26	0.11
SES	−0.07	0.05	−0.08	−0.04	0.05	−0.06	0.06	0.03	0.14*
ACEs	0.14	0.12	0.08	0.10	0.10	0.06	0.00	0.05	0.00
Ext	0.76	0.38	0.15*	0.01	0.32	0.00	0.02	0.18	0.01
Agr	−0.20	0.51	−0.03	0.37	0.43	0.06	1.27	0.24	0.36***
Csc	−0.61	0.47	−0.10	−0.53	0.40	−0.09	−0.15	0.22	−0.05
Ner	0.69	0.38	0.14	0.74	0.32	0.18*	0.14	0.18	0.06
Opn	0.59	0.47	0.09	0.06	0.40	0.01	−0.19	0.22	−0.06
*Step 2*	*R*^*2*^ = 0.07			*R*^*2*^ = 0.11			*R*^*2*^ = 0.18		
	*F*(10, 246) = 1.76, *ns*			*F*(10, 246) = 3.05**			*F*(10, 246) = 5.29***		
	*F*∆(5, 246) = 2.33*			*F*∆(5, 246) = 2.20, *ns*			*F*∆(5, 246) = 6.02***		
A. Sex	−0.31	0.47	−0.04	1.37	0.41	0.21***	0.7	0.23	0.18**
A. Age	0.21	0.12	0.11	0.11	0.11	0.06	−0.02	0.06	−0.02
P. Gender	0.17	0.55	0.02	−0.10	0.47	−0.01	0.34	0.26	0.09
SES	−0.10	0.05	−0.12	−0.05	0.05	−0.07	0.06	0.03	0.15*
ACEs	0.10	0.11	0.06	0.09	0.10	0.06	0.00	0.06	0.00
Ext	0.71	0.37	0.14	−0.01	0.32	0.00	0.03	0.18	0.01
Agr	0.33	0.53	0.05	0.58	0.45	0.09	1.18	0.25	0.33***
Csc	−0.51	0.47	−0.08	−0.42	0.41	−0.07	−0.21	0.23	−0.07
Ner	0.51	0.37	0.11	0.66	0.32	0.16*	0.18	0.18	0.07
Opn	0.70	0.47	0.10	0.06	0.40	0.01	−0.18	0.23	−0.05
Warmth	−0.20	0.12	−0.12	−0.07	0.10	−0.05	0.03	0.06	0.03
Hostility	0.36	0.14	0.18*	0.22	0.12	0.13	−0.12	0.07	−0.12
Control	0.02	0.12	0.01	−0.09	0.10	−0.06	0.06	0.06	0.07
*Step 3*	*R*^*2*^ = 0.12			*R*^*2*^ = 0.13			*R*^*2*^ = 0.19		
	*F*(13, 243) = 2.51**			*F*(13, 243) = 2.80***			*F*(13, 243) = 4.48***		
	*F*∆(3, 243) = 4.79**			*F*∆(3, 243) = 1.82, *ns*			*F*∆(3, 243) = 1.67, *ns*		

*Note*. The presented values are from all steps of the regression model (*N* = 257 dyads). The predictors were entered as follows: Step 1: Adolescent sex and age, parent gender, SES and ACEs, Step 2: Parent Big Five domains, Step 3: Parental Warmth, Hostility, and Control. *Ext* extraversion, *Agr* agreeableness, *Csc* conscientiousness, *Ner* neuroticism, *Opn* openness

*p < 0.05, **p < 0.01, ***p < 0.001

**Table 7:** Parent dark triad, context, and parenting as predictors of adolescent behavioral outcomes

	Externalizing			Internalizing			Prosocial		
Predictors	*B*	SE *B*	*β*	*B*	SE *B*	*β*	*B*	SE *B*	*β*
A. Sex	−0.31	0.48	−0.04	1.45	0.41	0.22***	0.58	0.23	0.15*
A. Age	0.15	0.12	0.08	0.08	0.10	0.05	−0.02	0.06	−0.02
P. Gender	0.15	0.49	0.02	−0.04	0.41	−0.01	0.77	0.24	0.20**
SES	−0.05	0.05	−0.06	−0.06	0.05	−0.08	0.05	0.03	0.12*
ACEs	0.22	0.11	0.12	0.14	0.10	0.09	−0.02	0.06	−0.02
*Step 1*	*R*^*2*^ = 0.02			*R*^*2*^ = 0.07			*R*^*2*^ = 0.08		
	*F*(5, 251) = 1.28, *ns*			*F*(5, 251) = 3.81**			*F*(5, 251) = 4.26***		
A. Sex	−0.37	0.48	−0.05	1.50	0.41	0.23	0.59	0.23	0.15**
A. Age	0.67	0.58	0.09	0.06	0.11	0.04***	−0.04	0.06	−0.04
P. Gender	0.17	0.13	0.09	−0.19	0.49	−0.03	0.36	0.27	0.09
SES	−0.06	0.05	−0.07	−0.06	0.05	−0.08	0.04	0.03	0.11
ACEs	0.20	0.12	0.11	0.11	0.10	0.07	−0.01	0.06	−0.01
Psy	−0.01	0.11	−0.01	0.04	0.10	0.04	−0.15	0.05	−0.25**
Mac	0.01	0.11	0.01	−0.02	0.09	−0.02	0.04	0.05	0.06
NNe	0.00	0.03	−0.01	0.05	0.03	0.13*	0.01	0.01	0.02
Ant	0.03	0.02	0.13	0.00	0.02	0.00	−0.02	0.01	−0.15
AgE	0.01	0.03	0.04	−0.01	0.02	−0.03	0.01	0.01	0.08
*Step 2*	*R*^*2*^ = 0.04			*R*^*2*^ = 0.09			*R*^*2*^ = 0.17		
	*F*(10, 246) = 1.11, *ns*			*F*(10, 246) = 2.39*			*F*(10, 246) = 4.89***		
	*F*∆(5, 246) = 1.00, *ns*			*F*∆(5, 246) = 1.01, *ns*			*F*∆(5, 246) = 5.49***		
A. Sex	−0.48	0.47	−0.06	1.40	0.41	0.21***	0.62	0.23	0.16**
A. Age	0.18	0.12	0.09	0.06	0.11	0.04	−0.04	0.06	−0.04
P. Gender	0.67	0.58	0.09	−0.11	0.50	−0.02	0.38	0.28	0.10
SES	−0.08	0.05	−0.09	−0.06	0.05	−0.09	0.05	0.03	0.12*
ACEs	0.19	0.12	0.11	0.12	0.10	0.08	−0.01	0.06	−0.01
Psy	−0.06	0.11	−0.05	0.02	0.10	0.02	−0.14	0.05	−0.23*
Mac	−0.07	0.11	−0.06	−0.07	0.09	−0.07	0.06	0.05	0.10
NNe	−0.02	0.03	−0.05	0.04	0.03	0.11	0.01	0.01	0.04
Ant	0.02	0.02	0.10	0.00	0.02	−0.02	−0.02	0.01	−0.13
AgE	0.02	0.03	0.07	0.00	0.02	−0.02	0.01	0.01	0.07
Warmth	−0.22	0.12	−0.13	−0.10	0.10	−0.07	0.01	0.06	0.01
Hostility	0.38	0.14	0.20**	0.24	0.12	0.14*	−0.14	0.07	−0.14*
Control	−0.02	0.12	−0.01	−0.13	0.10	−0.08	0.03	0.06	0.04
*Step 3*	*R*^*2*^ = 0.10			*R*^*2*^ = 0.12			*R*^*2*^ = 0.18		
	*F*(13, 243) = 2.11*			*F*(13, 243) = 2.46**			*F*(13, 243) = 4.19***		
	*F*∆(3, 243) = 5.34**			*F*∆(3, 243) = 2.61, *ns*			*F*∆(3, 243) = 1.79, *ns*		

*Note*. The presented values are from all steps of the regression model (*N* = 257 dyads). The predictors were entered as follows: Step 1: Adolescent sex and age, parent gender, SES and ACEs, Step 2: Parent Dark Triad domains, Step 3: Parental Warmth, Hostility, and Control. *Psy* psychopathy, *Mac* machiavellianism, *NNe* narcissistic neuroticism, *Ant* antagonism, *AgE* agentic Extraversion

*p < 0.05, **p < 0.01, ***p < 0.001

For both Study 1 and 2, separate analyses were conducted for each personality group, including the Big Five and Dark Triad. Due to the number of tests, the Benjamini–Hochberg (B–H) False Discovery Rate was used to control for family-wise error (Benjamini & Hochberg, 1995). Information regarding methods for determining sample size, all manipulations, and all measures in the study is presented above. There were no data exclusions.

## Results

### Descriptive Statistics

Descriptive statistics and reliability for all scales are presented in Table 1. The first author may be contacted for correlation tables.

### Study 1: Parental Personality and Context as Predictors of Parenting

#### Big Five and parental warmth

In the model of fathers, the final regression equation for warmth was significant (Table 2). Steps 2 and 3 added significant explained variance. Paternal conscientiousness was a positive predictor of warmth. According to B–H corrected *p*-values, this effect lost significance. There was a significant interaction effect between ACEs and paternal conscientiousness, *F*(3, 150) = 7.78, *p* < 0.001, *R*^*2*^ = 0.13; *F*∆(1, 150) = 6.43, *p* < 0.013 (Fig. 1SA, Supplementary Information [SI]). For fathers with 2 or less ACEs, paternal conscientiousness was positively associated with warmth, *b* = 0.10, *SE* = 0.03, *p* < 0.006. However, for fathers with 3 or more ACEs, this association was not significant, *b* = 0.01, *SE* = 0.06, *ns*.

In the model of mothers, the final regression equation for warmth was not significant (Table 3). No steps added significant explained variance.

#### Big Five and parental hostility

In the model of fathers, the final regression equation for hostility was significant (Table 2). Step 2 added significant explained variance. Paternal agreeableness was a negative predictor of hostility. According to B–H corrected *p*-values, agreeableness remained significant.

In the model of mothers, the final regression equation for hostility was significant (Table 3). Step 2 added significant explained variance. Maternal agreeableness was a negative predictor of hostility. According to B–H corrected *p*-values, maternal agreeableness remained significant.

#### Big Five and parental control

In the model of fathers, the final regression equation for control was not significant (Table 2). No steps added significant explained variance.

In the model of mothers, the final regression equation for control was not significant (Table 3). Step 1 added significant explained variance. There was a significant interaction effect between SES and maternal extraversion, *F*(3, 125) = 3.76, *p* < 0.013, *R*^*2*^ = 0.08; *F*∆(1, 125) = 6.97, *p* < 0.010 (Fig. 2S). For mothers with low SES (SES ≤ 11), maternal extraversion was negatively associated with control, *b* = −1.12, *SE* = 0.51, *p* < 0.032. For mothers with high SES (SES ≥ 25), maternal extraversion was positively associated with control, *b* = 1.15, *SE* = 0.52, *p* < 0.030.

#### Dark Triad and parental warmth

In the model of fathers, the final regression equation for warmth was significant (Table 4). Steps 2 and 3 added significant additional variance. Paternal ACEs and narcissistic neuroticism were negative predictors of warmth, whereas paternal agentic extraversion was a positive predictor of warmth. When B–H corrected *p*-values were considered, narcissistic neuroticism and agentic extraversion remained significant.

There was a significant interaction effect between ACEs and paternal Machiavellianism, *F*(3, 150) = 9.68, *p* < 0.001, *R*^*2*^ = 0.16; *F*∆(1, 150) = 11.07, *p* < 0.002 (Fig. 1SB). For fathers with 2 or less ACEs, paternal Machiavellianism was negatively associated with warmth, *b* = −0.02, *SE* = 0.01, *p* < 0.014. For fathers with 3 or more ACEs, the association between paternal Machiavellianism and warmth was not significant, *b* = 0.01, *SE* = 0.01, *ns*.

In the model of mothers, the final regression equation for warmth was significant (Table 5). Step 3 added explained variance. Maternal antagonism was a negative predictor of warmth. When B–H corrected *p*-values were considered, antagonism was not significant.

#### Dark Triad and parental hostility

In the model of fathers, the final regression equation for hostility was significant (Table 4). Step 2 added significant explained variance. Paternal psychopathy and agentic extraversion were positive and negative predictors of hostility, respectively. When B–H corrected *p*-values were considered, all effects lost significance.

In the model of mothers, the final regression equation for hostility was significant (Table 5). Step 2 added significant explained variance. Maternal Machiavellianism was a positive predictor of hostility. When B–H corrected *p*-values were considered, maternal Machiavellianism remained significant.

#### Dark Triad and parental control

In the model of fathers, the final regression equation for control was not significant (Table 4). No steps added significant explained variance.

In the model of mothers, the final regression equation for control was not significant (Table 5). Step 1 explained significant variance in maternal control, and SES was a positive individual predictor.

#### Alternate parenting models

As separate mother and father models may suffer from low statistical power, regression models predicting parenting behavior of the total parent sample (*N* = 283) were also run. Predictors were entered as follows: Parent gender, SES and ACEs in Step 1, parental personality in Step 2, and interactions (Personality × SES, Personality × ACEs) in Step 3. In the Big Five model, parent gender (i.e., being a mother), agreeableness, and conscientiousness were positive predictors of warmth; SES and agreeableness were positive and negative predictors of hostility, respectively; parent gender (i.e., being a mother), ACEs, and conscientiousness were positive predictors, and agreeableness was a negative predictor, of control. In the Dark Triad model, antagonism and agentic extraversion were negative and positive predictors of warmth, respectively; parent gender (i.e., being a mother), SES, narcissistic neuroticism and antagonism were positive predictors of hostility; parent gender (i.e., being a mother) was a positive predictor of control. Further details and regression tables are presented in SI.

### Study 2: Parental Personality, Context, and Parenting as Predictors of Adolescent Behavioral Outcomes

#### Big Five and adolescent externalizing behavior

The final regression equation for externalizing behavior was significant (Table 6). Steps 2 and 3 added significant explained variance. Parental hostility was a positive individual predictor of adolescent externalizing behavior. According to B–H corrected *p*-values, this effect lost significance.

#### Big Five and adolescent internalizing behavior

The final regression equation for internalizing behavior was significant (Table 6). No steps added significant explained variance. Girls were more likely to report higher internalizing behavior problems. Parental neuroticism was a positive individual predictor of adolescent internalizing behavior. According to B–H corrected *p*-values, only adolescent sex remained significant.

#### Big Five and adolescent prosocial behavior

The final regression equation for prosocial behavior was significant (Table 6). Steps 1 and 2 added significant explained variance. Adolescent sex, SES and parental agreeableness were positive predictors of adolescent prosocial behavior. Girls were more likely to report higher prosocial behavior. According to B–H corrected *p*-values, parental agreeableness and adolescent sex remained significant.

#### Dark Triad and adolescent externalizing behavior

The final regression equation for externalizing behavior was significant (Table 7). Step 3 added significant explained variance. Parental hostility was a positive predictor of adolescent externalizing behavior. When B–H corrected *p*-values were considered, parental hostility lost significance.

#### Dark Triad and adolescent internalizing behavior

The final regression equation for internalizing behavior was significant (Table 7). No steps added significant explained variance. Adolescent sex, such that girls reported higher, and parental hostility were positive predictors of internalizing behavior. When B–H corrected *p*-values were considered, adolescent sex remained significant.

#### Dark Triad and adolescent prosocial behavior

The final regression equation for prosocial behavior was significant (Table 7). Step 2 added significant explained variance. Adolescent sex and SES were positive predictors, and parental psychopathy and hostility were negative predictors of adolescent prosocial behavior. Girls reported higher prosocial behavior. When B–H corrected *p*-values were considered, these effects lost significance.

## Discussion

The influence of parental personality on adolescent behavioral outcomes may operate through both direct and indirect pathways, subject to family and environmental contexts. The current study extends the personality and parenting literature which, to date, has focused primarily on the Big Five (Achtergarde et al., 2015; Prinzie et al., 2009) and did not take into account the influence of contextual factors. Results indicate that a wider spectrum of parental personality domains, including the Big Five and Dark Triad, are important in understanding personality–parenting associations and adolescent behavioral strengths and difficulties. Further, contextual factors, such as SES and parents’ ACEs, have direct effects on parenting behavior and adolescent behavioral outcomes, but also moderate certain personality–parenting associations.

### The Wide Spectrum of Parental Personality

Of the Big Five traits, parental agreeableness was associated with lower paternal and maternal hostility. These findings indicate that agreeableness is a salient domain with regard to less negative parenting behavior, across genders. In a meta-analysis, parental agreeableness was positively linked to parental warmth, behavioral control, and autonomy support (Prinzie et al., 2009). Agreeableness represents aspects of behavior that are characterized by empathy, supportiveness, and flexibility (McCrae & Costa, 2008). Hence, agreeableness is a trait that facilitates effective parenting, especially during adolescence when parent–offspring interactions become more egalitarian and offspring desire greater autonomy (Noom et al., 2001).

Of the Dark Triad domains, mothers and fathers evinced varying patterns of personality–parenting associations. Narcissism domains were relevant for both maternal and paternal parenting, albeit in different ways. Agentic extraversion and narcissistic neuroticism positively and negatively predicted warmth, respectively, for fathers. Vulnerable narcissism, the larger personality domain of narcissistic neuroticism, has been linked to defensive and hostile interpersonal behavior (Sedikides, 2021). For mothers, antagonism was negatively associated with warmth. Previous work indicates maladaptive narcissism indirectly predicted less authoritative parenting through lack of empathy, whereas adaptive narcissism (e.g., authority, self-sufficiency) was associated with more adaptive parenting via higher empathy (Hart et al., 2017). Results of the current study support prior work such that narcissistic traits were associated with the warmth (i.e., empathic) dimension of parenting. Further evidence is provided regarding specific narcissistic traits, and findings suggest the presence of gender differences in how parents’ adaptive and maladaptive narcissistic traits influence their parenting behaviors.

The “darker” nature of Machiavellianism and psychopathy appears to extend to the use of negative parenting strategies. Both maternal and paternal Machiavellianism are associated with adolescent perceived parental rejection (Láng, 2018). Results indicate that, in addition to parental rejection, mothers high on Machiavellianism display hostile and aggressive parenting behaviors towards their offspring. Psychopathic traits have been linked to higher authoritarian parenting, characteristic of low warmth and high control (Cox et al., 2018). Paternal psychopathy was unassociated with paternal control in the current study, but was positively associated with hostility. Lack of empathy and guilt have been identified as core characteristics of psychopathy (Verschuere et al., 2018), and may have driven the association between paternal psychopathy and hostile parenting in the current study. Mothers high in Machiavellianism, and fathers high in psychopathy, may therefore utilize greater parental hostility with their adolescent offspring.

In considering the influence of parental personality on adolescent behavioral strengths and difficulties, parental agreeableness and psychopathy were positively and negatively associated with adolescent self-reported prosocial behavior, respectively. In accordance with social learning theory (Bandura, 1977), adolescents may model the behavior their parents display in their interactions with peers and other adults. Psychopathy and agreeableness are characterized by opposing levels of empathy, such that highly agreeable individuals display high empathy, whereas those with high psychopathy display a lack of empathy. Supportive interactions with parents have been tethered to the development of prosocial behavior in adolescence, such that these interactions foster empathy and constitute a behavioral model (Carlo & Padilla‐Walker, 2020). However, severe trait dispositions like high psychopathy are to a substantial degree heritable (Viding et al., 2005). A lack of prosocial behavior in adolescence may thus be early signs of a trend toward psychopathy in adulthood.

With regard to parental personality, results demonstrate that simply assessing the Big Five is not enough. Dark Triad domains were significant predictors of parenting behaviors and adolescent prosocial behavior. The examination of additional personality domains also underlined the relevance of contextual factors in regard to the parent–offspring relationship.

### The Influence of Context

This research offers insights into the influence of contextual factors on parental personality–parenting associations and in affecting adolescent behavioral outcomes. Prior work indicates that personality interacts with stress to predict behavioral coping, especially in individuals who are highly stress reactive or are exposed to numerous stressors (Connor-Smith & Flachsbart, 2007). A previous study conducted with mothers assessed ecological adversity, child difficulty, and maternal Big Five personality as predictors of parenting (Kochanska et al., 2012). High ecological adversity was negatively associated with observed positive parenting, measured as responsiveness and positive affect, but was unassociated with maternal observed or self-reported power assertion. The current study extends the literature on personality and context by examining SES and parents’ ACEs in both mothers and fathers and adopting a broad conceptualization of personality.

For mothers, SES interacted with extraversion in predicting parental control. Maternal extraversion was positively associated with maternal control in high SES contexts, but negatively associated in very low SES contexts. Mothers high in extraversion, then, exerted more control in environments characterized by greater social resources. Extraversion encompasses warmth, positive activity, and affiliation, but also ambition, assertiveness, and dominance (McCrae & Costa, 2008). An individual who has achieved a higher level of education and economic prosperity would likely embody more assertive and ambitious traits (Jonassaint et al., 2011). Therefore, highly extraverted mothers who have attained greater prosperity may tend to control their family interactions, but extraverted mothers who have considerably less social and economic resources may not.

Whereas SES was salient for maternal personality, ACEs were influential in the personality–parenting relationship for fathers. Fathers who experienced three or more ACEs self-reported slightly lower than average warmth regardless of their level of conscientiousness. Greater experience with childhood adversity, especially lack of emotional support, has been linked with poor attachment in adulthood, and may conduce to negative and disorganized parent–child interactions (Murphy et al., 2014). For fathers who experienced relatively fewer adverse experiences, conscientiousness may serve an adaptive function in relating to greater use of parental warmth with their children. However, it appears that conscientiousness is protective against stressors only to a certain extent.

A similar pattern was observed for paternal Machiavellianism and warmth in the case of ACEs. When fathers had experienced two or less ACEs, Machiavellianism was negatively associated with paternal warmth. However, fathers who had experienced three or more ACEs reported lower than average warmth regardless of their level of Machiavellianism. Machiavellianism has previously been identified as a maladaptive trait when processing stressful life events (Lyons et al., 2019). This pattern was replicated within the father–offspring relationship. In contexts of greater experience with childhood adversity, fathers did not report expressing high levels of warmth with their child even if they had low levels of Machiavellianism.

Results demonstrate that home and family context can influence typical parental personality–parenting associations. New evidence was provided as to how these associations might operate when considering personality domains beyond the Big Five, such as the Dark Triad. Further, gender differences were observed in how context influences parental personality–parenting associations. Inclusion of a wider spectrum of personality and contextual factors in parenting research offers greater insight into how personal and environmental stress influences the parent–adolescent relationship and, in turn, adolescent development.

### Limitations and Future Directions

The current study engaged in a comprehensive assessment of parental personality domains, using data from both parent and adolescent participants, and an economically diverse sample. Nonetheless, limitations must be acknowledged. As regression models contained many predictors, the modest sample size can result in Type 1 errors or false positives. To control for the possibility of chance findings, the B–H procedure was used for correcting *p*-values. This led to several effects losing significance. It is possible that these effects were spurious, or that with a larger sample they would emerge as stronger (Button et al., 2013). Further, the addition of interaction terms in regression models had very small effects on model error, as interaction effects are difficult to detect (Whisman & McClelland, 2005).

Only self-report of parental personality and parenting behavior was included, which is influenced by common-method variance (Podsakoff et al., 2003). This method of assessment is additionally susceptible to social desirability biases, as has been found in research with the Dark Triad (Kowalski et al., 2018). In this sample, parents self-reported relatively high levels of warmth, which can be influenced by idealization and denial of hurtful behavior (Rohner et al., 2005). However, adolescent self-report of behavior was obtained as certain personality traits may engender parents to perceive adolescent behavior as more or less negative than it is (Clark et al., 2017). Future work may use observational methods to assess parenting behaviors.

Parental personality, parenting, and adolescent behavior was not assessed longitudinally to determine directionality. Therefore, it may only be concluded that there are associations between parental personality, parenting behavior, and adolescent behavioral outcomes. Parental personality and offspring temperament traits interact to influence parenting behavior (Achtergarde et al., 2015). Perhaps adolescents with higher rates of externalizing behaviors evoke negative, hostile parenting from their parents. According to Belsky’s (1984) process model of parenting, parental psychological characteristics (e.g., personality) exert strong influences—both direct and indirect—compared to offspring characteristics (e.g., temperament) or context (e.g., SES). Longitudinal designs would parse out the direction of these effects. A final limitation is that parents’ ACEs were assessed retrospectively. However, behavioral and neuroimaging evidence suggests that individuals remember negative events with greater detail and accuracy (Kensinger, 2007).

## Conclusion

Parental personality, parenting behavior, and context are important factors which influence adolescent behavioral outcomes and future mental health. Extant research remained limited by continuing to focus solely on the Big Five in parents. Further, many studies did not take into account the context within which the parent–offspring relationship occurs. To address this gap, the current study examined intraindividual parental personality traits, interindividual parenting behaviors, and contextual factors in influencing adolescent behavioral strengths and difficulties. Thus, empirical evidence was provided consistent with the Tri-Directional Framework of Parental Personality and Offspring Outcomes. Both maternal and paternal Dark Triad domains were more relevant to parental warmth and hostility than the traditionally assessed Big Five. Also, SES and parents’ ACEs influenced maternal and paternal personality–parenting associations, respectively, and adolescent behavioral outcomes. Results have important implications for future personality and developmental research, suggesting that researchers consider a more broad spectrum of personality beyond the Big Five, the differential influences of mothers and fathers, and how different traits operate under varying environmental and family contexts to influence adolescent development.

## Supplementary information

Supplementary Information
