# Supplementary material for: A Tri-Directional Examination of Parental Personality, Parenting Behaviors, and Contextual Factors in Influencing Adolescent Behavioral Outcomes
Source: J Youth Adolesc. 2022 Apr 15;51(8):1536–51. doi: 10.1007/s10964-022-01602-8 (PMC9232422; doi:10.1007/s10964-022-01602-8)

**Supplementary Information**

**A Tri-Directional Examination of Parental Personality, Parenting Behaviors, and Contextual Factors in Influencing Adolescent Behavioral Outcomes**

**Supplementary Phase 1: Total Parent Models (*N* = 283)**

***Big Five and Parental Warmth***

In the model including both mothers and fathers, the final regression equation was significant (Table 1S). Steps 1 and 2 added significant explained variance. Parental agreeableness was a positive predictor of warmth, and gender was significant, such that mothers self-reported high warmth. An interaction effect between ACEs and agreeableness emerged as a joint predictor of warmth. According to B-H corrected *p*-values, the effects of parent gender and agreeableness remained significant.

***Big Five and Parental Hostility***

In the model including both mothers and fathers, the final regression equation was significant (Table 1S). Steps 1 and 2 added significant explained variance. SES and parental agreeableness were positive and negative predictors of hostility, respectively. According to B-H corrected *p*-values, the effects of SES and parental agreeableness remained significant.

***Big Five and Parental Control***

In the model including both mothers and fathers, the final regression equation was significant (Table 1S). Steps 1 and 2 added significant explained variance. Parent ACEs, gender, and conscientiousness were positive predictors, whereas parental agreeableness was a negative predictor of control. According to B-H corrected p-values, no effects would remain significant.

***Dark Triad and Parental Warmth***

In the model including both mothers and fathers, the final regression equation was significant (Table 2S). All steps added significant explained variance. ACEs, parental psychopathy, and antagonism were negative predictors, and parental agentic extraversion was a positive predictor of warmth. Interaction effects between ACEs and psychopathy and ACEs and Machiavellianism were joint predictors. When B-H corrected *p*-values were considered, the interaction effects between ACEs and Machiavellianism and ACEs and psychopathy remained significant.

***Dark Triad and Parental Hostility***

In the model including both mothers and fathers, the final regression equation was significant (Table 2S). All steps added significant explained variance. Gender was significant, such that mothers reported using more hostility. SES, parental Machiavellianism, and narcissistic neuroticism were positive predictors of hostility. An interaction effect emerged between SES and narcissistic neuroticism. According to B-H corrected *p*-values, SES, parental narcissistic neuroticism, Machiavellianism, and the interaction between SES and narcissistic neuroticism would remain significant.

***Dark Triad and Parental Control***

In the model including both mothers and fathers, the final regression equation was significant (Table 2S). Step 1 added significant explained variance. Parent gender was a significant individual predictor, such that mothers reported higher control. According to B-H corrected *p*-values, parent gender would lose significance.

|  | Warmth | | | Hostility | | | Control | | |
| --- | --- | --- | --- | --- | --- | --- | --- | --- | --- |
| Predictors | *B* | SE *B* | *β* | *B* | SE *B* | *β* | *B* | SE *B* | *β* |
| SES | 0.00 | 0.00 | 0.05 | 0.01 | 0.00 | 0.13* | 0.06 | 0.03 | 0.12* |
| ACEs | -0.01 | 0.01 | -0.09 | 0.02 | 0.01 | 0.16** | 0.12 | 0.06 | 0.12* |
| Gender | 0.15 | 0.03 | 0.26*** | 0.00 | 0.03 | -0.01 | 0.88 | 0.25 | 0.20*** |
| **Step 1** | *R^2^* = .07 | | | *R^2^* = .04 | | | *R^2^* = .07 | | |
|  | *F*(3, 279) = 7.49*** | | | *F*(3, 279) = 3.58* | | | *F*(3, 279) = 7.51*** | | |
| SES | 0.00 | 0.00 | 0.02 | 0.01 | 0.00 | 0.16** | 0.06 | 0.03 | 0.13* |
| ACEs | -0.01 | 0.01 | -0.06 | 0.01 | 0.01 | 0.11 | 0.12 | 0.06 | 0.11 |
| Gender | 0.13 | 0.03 | 0.23*** | 0.02 | 0.03 | 0.03 | 0.81 | 0.28 | 0.19** |
| Ext | 0.00 | 0.03 | 0.00 | 0.01 | 0.02 | 0.04 | 0.07 | 0.20 | 0.02 |
| Agr | 0.10 | 0.03 | 0.18** | -0.17 | 0.03 | -0.33*** | -0.65 | 0.27 | -0.16* |
| Csc | 0.06 | 0.03 | 0.13* | -0.01 | 0.03 | -0.03 | 0.58 | 0.25 | 0.16* |
| Ner | -0.02 | 0.02 | -0.06 | 0.04 | 0.02 | 0.11 | 0.14 | 0.20 | 0.05 |
| Opn | 0.02 | 0.03 | 0.03 | -0.03 | 0.03 | -0.06 | -0.42 | 0.25 | -0.11 |
| **Step 2** | *R^2^* = .17 | | | *R^2^* = .20 | | | *R^2^* = .12 | | |
|  | *F*(8, 274) = 6.79*** | | | *F*(8, 274) = 8.34*** | | | *F*(8, 274) = 4.58*** | | |
|  | *F*$\Delta$(5, 274) = 5.97*** | | | *F*$\Delta$(5, 274) = 10.66*** | | | *F*$\Delta$(5, 274) = 2.67* | | |
| SES | 0.00 | 0.00 | 0.02 | 0.01 | 0.00 | 0.14* | 0.05 | 0.03 | 0.10 |
| ACEs | -0.01 | 0.01 | -0.09 | 0.01 | 0.01 | 0.11 | 0.14 | 0.07 | 0.14* |
| Gender | 0.12 | 0.04 | 0.21*** | 0.02 | 0.03 | 0.03 | 0.84 | 0.29 | 0.19** |
| Ext | 0.00 | 0.03 | 0.00 | 0.01 | 0.02 | 0.03 | 0.07 | 0.20 | 0.02 |
| Agr | 0.09 | 0.03 | 0.18** | -0.17 | 0.03 | -0.32*** | -0.65 | 0.27 | -0.16* |
| Csc | 0.07 | 0.03 | 0.16* | -0.01 | 0.03 | -0.02 | 0.55 | 0.25 | 0.15* |
| Ner | -0.02 | 0.03 | -0.05 | 0.04 | 0.03 | 0.11 | 0.12 | 0.21 | 0.05 |
| Opn | 0.01 | 0.03 | 0.02 | -0.03 | 0.03 | -0.07 | -0.41 | 0.25 | -0.11 |
| Ext x SES | 0.00 | 0.01 | 0.02 | 0.00 | 0.01 | 0.01 | 0.09 | 0.05 | 0.14 |
| Ext x ACEs | 0.00 | 0.01 | -0.01 | 0.00 | 0.01 | 0.00 | 0.05 | 0.09 | 0.04 |
| Agr x SES | 0.01 | 0.01 | 0.12 | -0.01 | 0.01 | -0.07 | -0.05 | 0.06 | -0.06 |
| Agr x ACEs | 0.03 | 0.02 | 0.12 | -0.01 | 0.02 | -0.02 | -0.07 | 0.17 | -0.03 |
| Csc x SES | 0.00 | 0.01 | -0.01 | 0.01 | 0.01 | 0.07 | 0.02 | 0.06 | 0.03 |
| Csc x ACEs | -0.02 | 0.02 | -0.11 | -0.01 | 0.02 | -0.04 | 0.02 | 0.13 | 0.01 |
| Ner x SES | 0.00 | 0.01 | 0.02 | 0.01 | 0.01 | 0.09 | 0.01 | 0.04 | 0.02 |
| Ner x ACEs | 0.01 | 0.01 | 0.04 | 0.00 | 0.01 | 0.00 | -0.09 | 0.08 | -0.09 |
| Opn x SES | 0.01 | 0.01 | 0.07 | 0.00 | 0.01 | 0.00 | -0.11 | 0.06 | -0.11 |
| Opn x ACEs | 0.01 | 0.01 | 0.05 | 0.01 | 0.01 | 0.07 | -0.13 | 0.12 | -0.08 |
| **Step 3** | *R^2^* = .20 | | | *R^2^* = .21 | | | *R^2^* = .15 | | |
|  | *F*(18, 264) = 3.59*** | | | *F*(18, 264) = 3.99*** | | | *F*(18, 264) = 2.49*** | | |
|  | *F*$\Delta$(10, 264) = 1.02, *ns* | | | *F*$\Delta$(10, 264) = 0.61, *ns* | | | *F*$\Delta$(10, 264) = .59, *ns* | | |

**Table 1S**

*Parent Big Five Domains and Contextual Factors as Predictors of Parenting*

Note. Presented values are from all steps of the hierarchical regression analyses (*N* = 283). Predictors were entered as follows: Step 1: Gender, SES, and ACEs, Step 2: Parent Big Five domains, Step 3: The five interaction terms of SES and Big Five and five interaction terms of ACEs and Big Five. Ext = Extraversion, Agr = Agreeableness, Csc = Conscientiousness, Ner = Neuroticism, Opn = Openness.

^ *p* < .10, * *p* < .05, ** *p* < .01, *** *p* < .001

**Table 2S**

*Parent Dark Triad Domains and Contextual Factors as Predictors of Parenting*

|  | Warmth | | | Hostility | | | Control | | |
| --- | --- | --- | --- | --- | --- | --- | --- | --- | --- |
| Predictors | *B* | SE *B* | *β* | *B* | SE *B* | *β* | *B* | SE *B* | *β* |
| SES | 0.00 | 0.00 | 0.05 | 0.01 | 0.00 | 0.13* | 0.06 | 0.03 | 0.12* |
| ACEs | -0.01 | 0.01 | -0.09 | 0.02 | 0.01 | 0.16** | 0.12 | 0.06 | 0.12* |
| Gender | 0.15 | 0.03 | 0.26*** | 0.00 | 0.03 | -0.01 | 0.88 | 0.25 | 0.20*** |
| **Step 1** | *R^2^* = .07 | | | *R^2^* = .04 | | | *R^2^* = .07 | | |
|  | *F*(3, 279) = 7.49*** | | | *F*(3, 279) = 3.58* | | | *F*(3, 279) = 7.51*** | | |
| SES | 0.00 | 0.00 | 0.04 | 0.01 | 0.00 | 0.15* | 0.06 | 0.03 | 0.12* |
| ACEs | 0.00 | 0.01 | -0.02 | 0.01 | 0.01 | 0.06 | 0.09 | 0.06 | 0.09 |
| Gender | 0.08 | 0.04 | 0.14* | 0.08 | 0.04 | 0.15* | 0.91 | 0.30 | 0.21** |
| Psy | -0.01 | 0.01 | -0.11 | 0.01 | 0.01 | 0.16 | 0.06 | 0.06 | 0.09 |
| Mac | -0.01 | 0.01 | -0.13 | 0.01 | 0.01 | 0.14 | 0.04 | 0.06 | 0.06 |
| NNe | 0.00 | 0.00 | -0.10 | 0.01 | 0.00 | 0.17** | 0.03 | 0.02 | 0.10 |
| Ant | 0.00 | 0.00 | -0.16 | 0.00 | 0.00 | 0.18* | -0.01 | 0.01 | -0.08 |
| AgE | 0.00 | 0.00 | 0.13* | 0.00 | 0.00 | -0.10 | 0.00 | 0.01 | -0.02 |
| **Step 2** | *R^2^* = .17 | | | *R^2^* = .19 | | | *R^2^* = .09 | | |
|  | *F*(8, 274) = 7.26*** | | | *F*(8, 274) = 8.25*** | | | *F*(8, 274) = 3.57*** | | |
|  | *F*$\Delta$(5, 274) = 6.87*** | | | *F*$\Delta$(5, 274) = 10.67*** | | | *F*$\Delta$(5, 274) = 1.17, *ns* | | |
| SES | 0.00 | 0.00 | 0.03 | 0.01 | 0.00 | 0.12* | 0.05 | 0.03 | 0.10 |
| ACEs | -0.02 | 0.01 | -0.13 | 0.01 | 0.01 | 0.09 | 0.08 | 0.07 | 0.08 |
| Gender | 0.06 | 0.04 | 0.11 | 0.08 | 0.04 | 0.14* | 0.87 | 0.31 | 0.20** |
| Psy | -0.01 | 0.01 | -0.12 | 0.01 | 0.01 | 0.16 | 0.05 | 0.06 | 0.07 |
| Mac | -0.01 | 0.01 | -0.13 | 0.01 | 0.01 | 0.15 | 0.06 | 0.06 | 0.08 |
| NNe | 0.00 | 0.00 | -0.08 | 0.01 | 0.00 | 0.15** | 0.03 | 0.02 | 0.10 |
| Ant | 0.00 | 0.00 | -0.20* | 0.00 | 0.00 | 0.18* | -0.01 | 0.01 | -0.09 |
| AgE | 0.00 | 0.00 | 0.15* | 0.00 | 0.00 | -0.11 | 0.00 | 0.01 | -0.02 |
| Psy x SES | 0.00 | 0.00 | 0.01 | 0.00 | 0.00 | -0.15 | -0.01 | 0.01 | -0.10 |
| Psy x ACEs | -0.01 | 0.00 | -0.21* | 0.00 | 0.00 | -0.06 | -0.01 | 0.04 | -0.03 |
| Mac x SES | 0.00 | 0.00 | -0.05 | 0.00 | 0.00 | 0.04 | 0.00 | 0.01 | 0.01 |
| Mac x ACEs | 0.01 | 0.00 | 0.32*** | 0.00 | 0.00 | -0.05 | 0.04 | 0.03 | 0.13 |
| NNe x SES | 0.00 | 0.00 | -0.05 | 0.00 | 0.00 | 0.10 | 0.00 | 0.00 | 0.01 |
| NNe x ACEs | 0.00 | 0.00 | 0.01 | 0.00 | 0.00 | 0.03 | 0.00 | 0.01 | -0.04 |
| Ant x SES | 0.00 | 0.00 | -0.08 | 0.00 | 0.00 | 0.16 | 0.00 | 0.00 | 0.13 |
| Ant x ACEs | 0.00 | 0.00 | -0.11 | 0.00 | 0.00 | 0.12 | -0.01 | 0.01 | -0.10 |
| AgE x SES | 0.00 | 0.00 | 0.07 | 0.00 | 0.00 | -0.03 | 0.00 | 0.00 | -0.04 |
| AgE x ACEs | 0.00 | 0.00 | -0.05 | 0.00 | 0.00 | 0.00 | 0.00 | 0.01 | 0.02 |
| **Step 3** | *R^2^* = .23 | | | *R^2^* = .22 | | | *R^2^* = .11 | | |
|  | *F*(18, 264) = 4.37*** | | | *F*(18, 264) = 4.20*** | | | *F*(18, 264) = 1.88* | | |
|  | *F*$\Delta$(10, 264) = 1.87* | | | *F*$\Delta$(10, 264) = 0.97, *ns* | | | *F*$\Delta$(10, 264) = 0.57, *ns* | | |

Note. Presented values are from all steps of the hierarchical regression analyses (*N* = 283). Predictors were entered as follows: Step 1: SES and ACEs, Step 2: Parent Dark Triad domains, Step 3: The five interaction terms of SES and Dark Triad and five interaction terms of ACEs and Dark Triad. Psy = Psychopathy, Mac = Machiavellianism, NNe = Narcissistic Neuroticism, Ant = Antagonism, AgE = Agentic Extraversion.

* *p* < .05, ** *p* < .01, *** *p* < .001

**Figure 1S**

A. *ACEs Moderate the Association Between Paternal Conscientiousness and Warmth*, *F*(3, 150) = 7.78, *p* < .001, *R^2^* = 0.13; *F*$\Delta$(1, 150) = 6.43, *p* < .013. *Two ACEs*: *b* = 0.10, *SE* = 0.03, *p* < .006; *Four ACEs*: *b* = 0.01, *SE* = 0.06, *ns*.

B. *ACEs Moderate the Association Between Paternal Machiavellianism and Warmth*, *F*(3, 150) = 9.68, *p* < .001, *R^2^* = 0.16; *F*$\Delta$(1, 150) = 11.07, *p* < .002. *Two ACEs*: *b* = -0.02, *SE* = 0.01, *p* < .014; *Four ACEs*: *b* = 0.01, *SE* = 0.01, *ns*.


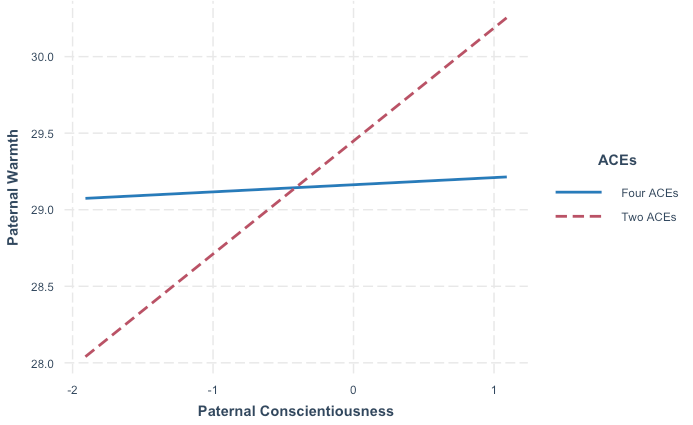
A.


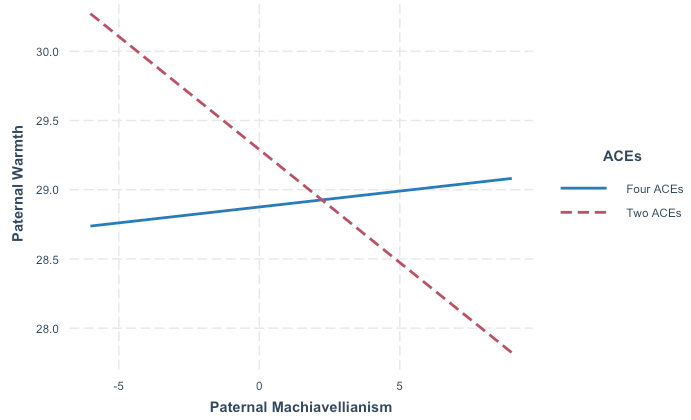


B.

**Figure 2S**

*SES Moderates the Association Between Maternal Extraversion and Control*, *F*(3, 125) = 3.76, *p* < .013, *R^2^* = 0.08; *F*$\Delta$(1, 125) = 6.97, *p* < .010. *Low SES*: *b* = -1.12, *SE* = 0.51, *p* < .032; *High SES*: *b* = 1.15, *SE* = 0.52, *p* < .030.


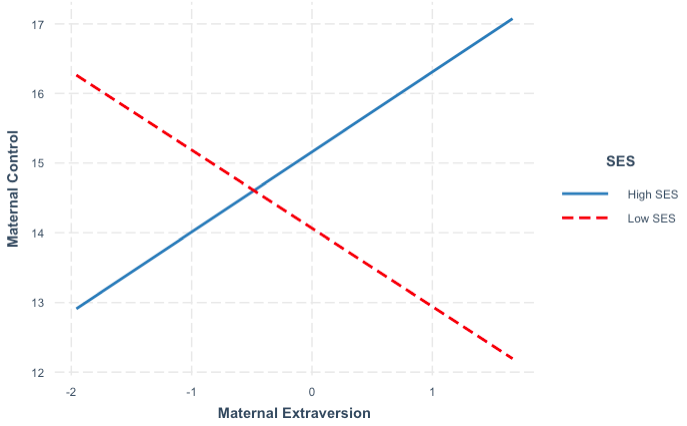

Supplement: Supplementary file 1 — Supplementary Information [file 10964_2022_1602_MOESM1_ESM.docx]
